# Pre-sarcopenia is the prognostic factor of overall survival in early-stage hepatoma patients undergoing radiofrequency ablation

**DOI:** 10.1097/MD.0000000000020455

**Published:** 2020-06-05

**Authors:** Wen-Shuo Yeh, Pi-Ling Chiang, Kwong-Ming Kee, Ching-Di Chang, Sheng-Nan Lu, Chien-Hung Chen, Jing-Houng Wang

**Affiliations:** a Division of Hepatogastroenterology, Department of Internal Medicine; b Department of Diagnostic Radiology, Kaohsiung Chang Gung Memorial Hospital, and Chang Gung University College of Medicine, Kaohsiung City; c Division of Hepatogastroenterology, Department of Internal Medicine, Chiayi Chang Gung Memorial Hospital, Chiayi County, Taiwan.

**Keywords:** hepatocellular carcinoma, mortality, radiofrequency ablation, recurrence, sarcopenia

## Abstract

Sarcopenia might have impact on the outcome of patients with hepatoma carcinoma (HCC). This study *was to determine whether pre-sarcopenia is associated with the outcome* of HCC patients undergoing radiofrequency ablation (RFA).

Patients with newly diagnosed HCC undergoing RFA were enrolled. We excluded patients without pre-RFA abdominal computed tomography or with incomplete ablation. Psoas muscle area index was calculated at the mid-lumbar 3 level of computed tomography images with the manual trace method. Pre-sarcopenia was defined as psoas muscle area index less than 4.24 and 2.50 cm^2^/m^2^ for males and females respectively. The demographics and clinical characteristics were recorded before RFA.

All patients were followed regularly until death or end of 2018. A total of 136 patients, including – BCLC stage 0 (n = 44, 32.4%) and – stage A (n = 92, 67.6%), were enrolled (males/females: 78/58, age: 65.4 years) with a mean follow-up period of 3.84 years. There were 75 patients (55.1%) with HCC recurrence and 47 patients (34.6%) with mortality during follow-up. Twenty-two (16.2%) patients were diagnosed with pre-sarcopenia. Multivariate analysis showed pre-sarcopenia (HR: 2.110 (1.092–4.078); *P* = .026) was the only factor significantly associated with overall survival (OS); however, there were no factors associated with HCC recurrence.

For patients without and with pre-sarcopenia, the 1-, 3-, and 5-year OS rates were 92.0%, 77.6%, 68.9%, and 81.8%, 54.5%, 44.1% respectively (*P* = .007). For early-stage HCC patients undergoing RFA, pre-sarcopenia is the prognostic factor of OS, but not of recurrence, with a worse 5-year OS rate of 44.1%.

## 1 Introduction

East and southeast Asia have the highest rates of liver cancer compared to other regions. Hepatocellular carcinoma (HCC) is the predominant primary liver cancer occurring worldwide.^[1]^ Curative treatments including surgical resection or radiofrequency ablation (RFA) have been used to treat early-stage HCC.^[2]^ In a previous literature review, the rate of tumor recurrence was 58% to 81% at 5 years and overall survival was 40% to 68% at 5 years after RFA for HCC within Milan criteria.^[3]^ To prolong patient survival and reduce tumor recurrence, it might be important to improve host factors in addition to adjusting HCC treatment algorithms or modalities.

Sarcopenia is a geriatric syndrome that is associated with the decline of skeletal muscle mass and muscle strength.^[4]^ Pre-sarcopenia means patients demonstrate evidence of decline of skeletal muscle mass. The cause of sarcopenia is multifactorial,^[5]^ and is associated with aging, multi-morbidity, and chronic diseases such as type 2 diabetes, chronic kidney disease, and cancer. *The incidence of sarcopenia, known as primary sarcopenia, increases with age.* Liver cirrhosis or HCC can cause secondary sarcopenia due to inflammation, dysfunction of protein anabolism, malnutrition.^[6]^ In clinical practice, computed tomography (CT) has been widely used to evaluate the stage of HCC and to measure the skeletal muscle mass in HCC patients.^[7–9]^ Several clinical or biochemical predictors have been found to predict clinical outcomes for HCC patients who have undergone RFA,^[10]^ although few studies have mentioned the relationship between skeletal muscle loss and the outcome of patients with HCC treated with RFA. One meta-analysis report indicated that patients with sarcopenia or pre-sarcopenia had poor outcomes after treatment.^[9]^ However, the relationship between sarcopenia and the prognosis of HCC patients undergoing RFA is inconsistent and populations appear heterogeneous among studies.^[7,8,11–13]^ The aims of this study were to investigate the impact of pre-sarcopenia and other clinical factors on survival and recurrence for patients with very early- or early-stage HCC undergoing RFA.

## 2 Materials and methods

### 2.1 Patients

We retrospectively evaluated patients with naïve HCC between January 2012 and December 2013. We enrolled patients with Barcelona clinic liver cancer (BCLC) stage 0 and A who had undergone percutaneous RFA alone as initial HCC therapy. Patients without complete therapy were excluded from this study, as were patients without abdominal CT scan before RFA therapy. HCC was diagnosed according to the radiological and/or histological criteria recommended from the guidelines.^[14]^ Associated prognostic factors were collected to represent the severity of liver fibrosis and liver function respectively. The protocol of this study was approved by the Institutional Review Board of Chang Gung Memorial Hospital, Taiwan (201900455B0).

### 2.2 Assessment of psoas muscle mass

**Figure 1:**
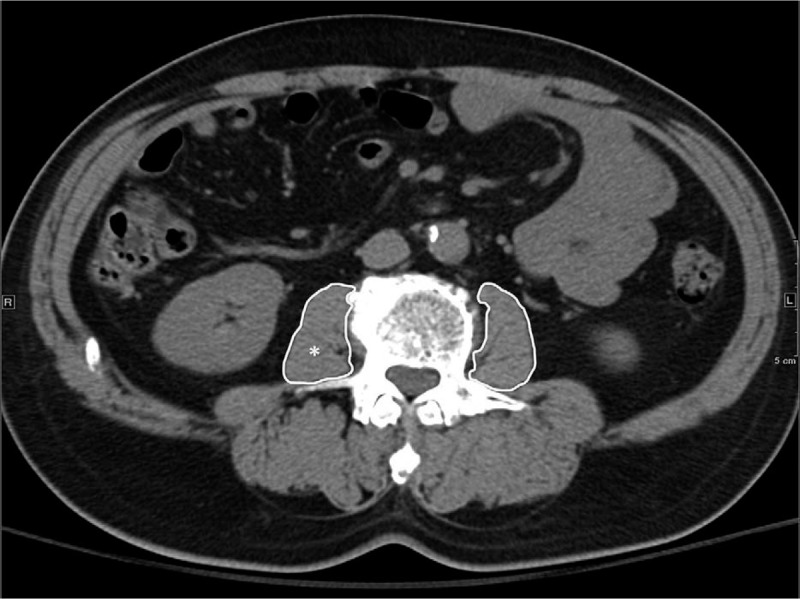
Measurement of psoas muscle area index. Psoas muscle area index [psoas muscle area (^∗^) at the mid-L3 level in CT (cm^2^)/height (m^2^)] was calculated by computed tomography within 3 months before RFA treatment with the manual trace method.

To assess skeletal muscle mass, we used the psoas muscle area index (PMI) to measure the volume of muscle mass before treatment. The pre-treatment PMI [psoas muscle area at the mid-L3 level in CT (cm^2^)/height (m^2^)] was calculated by CT within 3 months before RFA treatment with the manual trace method. PMI is a simple method to evaluate the pre-sarcopenia condition, and was used as a parameter to evaluate the skeletal muscle mass of the whole body. According to the previous study, the cut-off values of PMI for pre-sarcopenia was 4.24 cm^2^/m^2^ for males and 2.50 cm^2^/m^2^ for females.^[15]^Figure 1 shows the method used to measure PMI in patients with potential pre-sarcopenia in our study.

### 2.3 Techniques of RFA

RFA was performed on in-patients by using a Cool-Tip RFA electrode (Radionics, Burlington, MA), Big-Tip electrode (RF Medical Co., Ltd., Korea) or Viva RF electrode system (STARmed, Korea). Procedures were performed percutaneously using real-time sonographic guidance on patients by experienced hepatologists who had over 5 years of experience. For large tumors, multiple overlapping ablations were required so that each tumor could reach an ablation zone extending 0.5 cm beyond the tumor margin. After each ablation in the tumor, the electrode was stepwise pulled out with ablation from the insertion site under the tip temperature measuring more than 80°C to reduce the risk of bleeding and tumor seeding. All patients were hospitalized for more than 1 day to monitor any post-RFA complications.

### 2.4 Follow-up

Complete ablation was confirmed by radiologic findings showing absence of contrast enhancement of all lesions within 1 month after RFA. After curative treatment, we regularly followed up the level of alpha-fetoprotein , abdominal ultrasounds or abdominal CT per every 3 to 6 months. When recurrent HCC was suspected, further examinations such as dynamic CT or liver MRI would be performed. All patients were followed until the end of the study in November 2018 or death. The primary endpoints of this study were overall survival (OS) and HCC recurrence. Survival and recurrence data were obtained from the clinical medical chart.

## 3 Statistical analysis

**Figure 2:**
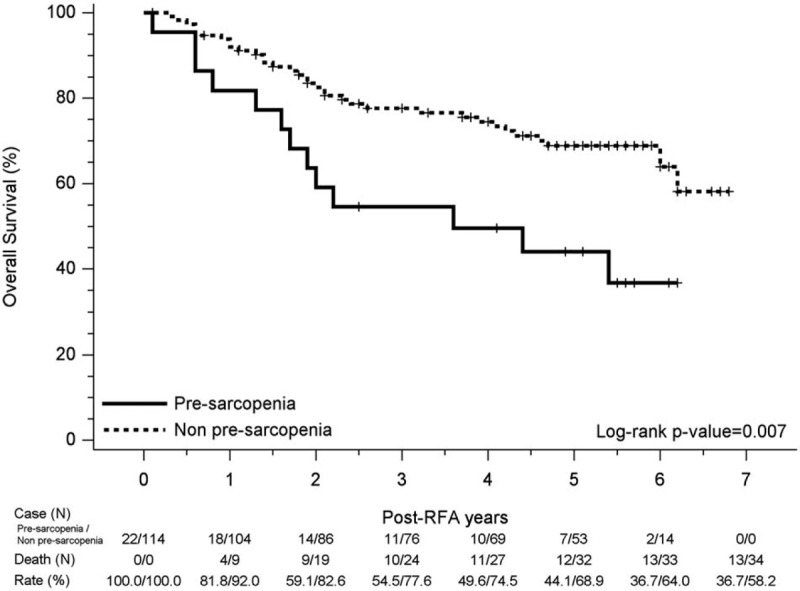
Kaplan–Meier curve for overall survival in pre-sarcopenia and non-pre-sarcopenia group. Significant reduced overall survival was observed in the pre-sarcopenia group, of which 1-, 3-, and 5-year cumulative OS rates were 81.8%, 54.5%, 44.1% compared to the non-pre-sarcopenia group, of which 1-, 3-, and 5-year cumulative overall survival rates were 92.0%, 77.6%, 68.9%; log-rank *P* value = .007.

**Figure 3:**
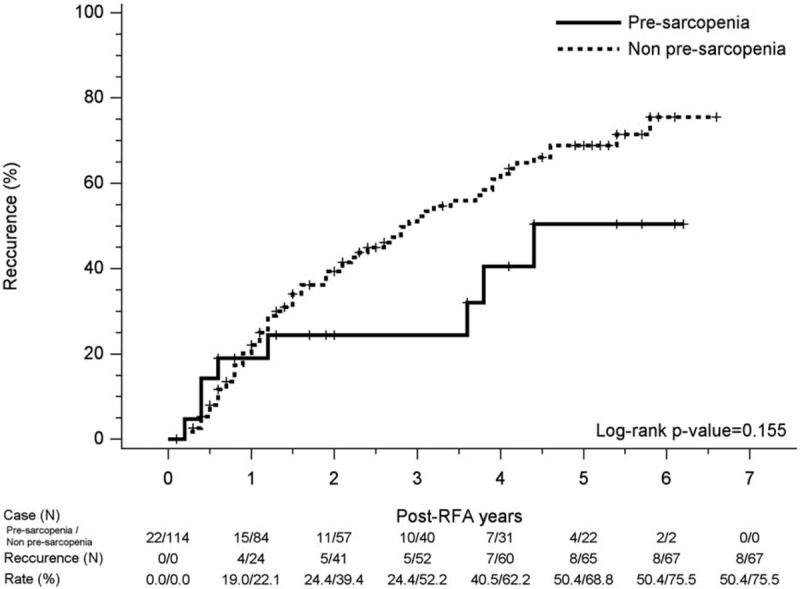
Kaplan–Meier curve for recurrence hepatoma carcinoma events in pre-sarcopenia and non-pre-sarcopenia group. There was no significant difference in recurrence rate between the 2 groups.

Associated continuous prognostic factors with relevant outcomes were evaluated using the Student *t* test, with associated categorical parameters with relevant outcome being evaluated using Fisher exact test or Chi-squared test. Independent prognostic factors associated with recurrence or survival were determined by Cox proportional hazard model, while OS and recurrence curves were evaluated using the Kaplan-Meier method and the difference between the curves was tested by using the log–rank test. Figures 2 and 3 were made by SigmaPlot version 12.3, from Systat Software, Inc., San Jose California, CA. The statistical analyses were performed using the Statistical Package for Social Sciences (SPSS, version 22, SPSS Inc. Armonk, NY). The statistical significance was set at P < .05.

## 4 Results

### 4.1 Patients

**Figure 4:**
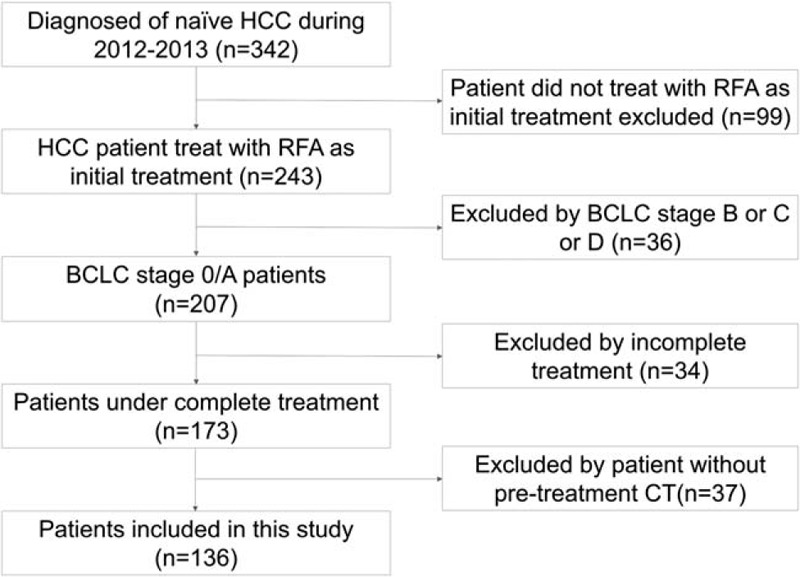
Flow chart of patient enrollment.

A total of 136 patients (males/females: 78/58; mean age: 63.4 ± 10.1 years), including in BCLC stage 0 (n = 44, 32.4%) and stage A (n = 92, 67.6%), were enrolled. Figure 4 shows the flow chart of enrollment in this study. Twenty-two (16.2%) patients had pre-sarcopenia by pre-treatment CT. The most etiologies of HCC in these patients were hepatitis B virus (n = 50, 36.8%), hepatitis C virus (n = 74, 54.4%), and alcohol drinking (n = 38, 27.9%). The mean of tumor sizes was 2.16 ± 0.71 cm (range from 1.1  to 4.5 cm). Most of the tumor number was solitary (75.7%) and the maximum tumor number was 3. Baseline demographic and clinical data of patients with and without pre-sarcopenia are shown in Table 1. The mean follow-up duration was 3.84 ± 1.98 years. Seventy-five patients (55.1%) had recurrence HCC and 47 patients (34.6%) expired during follow-up. The mean duration between pre-treatment CT and RFA was 36.7 ± 24.1 days. Patients with pre-sarcopenia had lower body mass index than those without, and had more proportion of grade 2 and grade 3 of albumin to bilirubin grade , as well as proportions of alpha-fetoprotein over 20 ng/mL in these patients; finally, the pre-treatment platelet was lower in this group than in the non-pre-sarcopenia group. The patient's age, gender, tumor size, tumor number, underlying disease, fibrosis-4, albumin, prothrombin time, liver enzyme, total bilirubin showed no differences between the 2 groups.

**Table 1:**
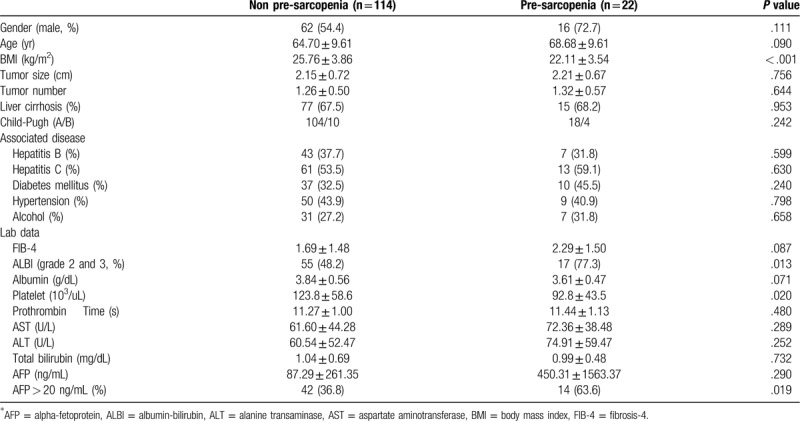
Demographic characteristics of hepatoma carcinoma patients with or without pre-sarcopenia.

### 4.2 Overall survival

Table 2 shows the prognostic factors for OS on univariate and multivariate analysis. Overall survival rates of patients with HCC were significantly affected by Child-Pugh B (HR: 2.406 (1.117–5.181); *P* = .025), pre-sarcopenia patients (HR: 2.338 (1.232–4.436); *P* = .009), high grade of albumin to bilirubin grade (HR: 2.074 (1.132–3.798); *P* = .018), a-fetoprotein > 20 (HR: 2.027 (1.166–3.697); *P* = .013) according to univariate analysis. Pre-sarcopenia (HR: 2.472 (1.299–4.703); *P* = .06) was the only independent factor in poor OS rate in multivariable Cox regression analysis.

**Table 2:**
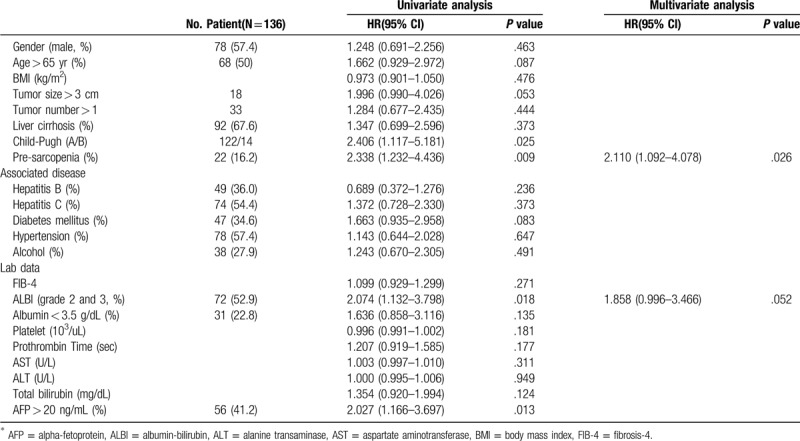
Prognostic factors for overall survival on univariate and multivariate analysis.

For all patients, the 1-, 3-, and 5-year cumulative OS rates were 90.3%, 73.7%, and 64.7%, respectively. Reduced OS rates were observed in the pre-sarcopenia group, of which the 1-, 3-, and 5-year cumulative OS rates were 81.8%, 54.5%, 44.1% compared to those without pre-sarcopenia, (1-, 3-, and 5-year cumulative OS rates of 92.0%, 77.6%, 68.9%, respectively; Log-Rank *P* value = .007) (Fig. 2).

### 4.3 Recurrence rate

For all patients, the 1-, 3-, and 5-year cumulative recurrence rates were 21.6%, 48.3%, and 66.3% respectively. Figure 3 shows the Kaplan–Meier curve for recurrent HCC events in pre-sarcopenia and without pre-sarcopenia groups, with no significant difference in recurrence rate between the 2 groups. There were no significant prognostic factors associated with HCC recurrence in multivariate analysis.

### 4.4 Discussion and conclusions

Several prognostic factors of patients with HCC who had undergone RFA have been shown in previous studies.^[10]^ However, few studies have shown the relationship of pre-sarcopenia and the outcomes in patients with naïve HCC who underwent RFA. In patients with very early- or early-stage HCC in this study, there were 16.2% patients with pre-sarcopenia. Our study revealed that pre-sarcopenia is the independent factor associated with overall survival in patients with naïve HCC who underwent RFA. However, pre-sarcopenia was not the factor associated with HCC recurrence in this study.

Previous studies reported that the prevalence of sarcopenia in HCC patients ranged from 12.4% to 66.3%.^[9]^ For diagnosis of sarcopenia, muscle strength and muscle mass should be measured. However, most studies were retrospective and did not measure muscle strength at initial HCC diagnosis. According to the international consensus, the cut-off value of skeletal muscle depletion is set at 2 standard deviations below the mean value of a young reference group. Asian studies have reported a lower prevalence of sarcopenia through this approach than Caucasian studies because of some cohort effect.^[5]^ Older Asian people today may have performed more physical activity in agricultural and traditional lifestyles when they were young, so their muscle mass may be maintained better than that of the younger generation. The prevalence of sarcopenia in our study was lower than the mean of other studies. This might be due to most of our patients in south-western Taiwan having traditional lifestyles and doing farming work in their early adulthood.

Skeletal muscle depletion is a strong prognostic factor for overall survival in the patients diagnosed with different cancers.^[16]^ One meta-analysis reported that sarcopenia showed poor overall survival in patients with HCC receiving different therapies.^[9]^ The pathophysiology of sarcopenia and poor overall survival in patients with HCC who have undergone therapy is indeterminate. One major cause of sarcopenia and fragility is the alteration of the endocrine system involved in the inflammatory processes, muscle synthesis, and protein catabolism.^[17]^ Decrease of insulin-like growth factor-1 (IGF-1) is strongly associated with low muscle mass and muscle strength.^[18]^ Patients with HCC have lower IGF-1 levels than those with cirrhosis or healthy people. One study reported that lower plasma IGF-1 levels in patients with HCC correlated with poor overall survival.^[19]^ Even though the mechanism of sarcopenia is multifactorial, it might imply that lower IGF-1 and severe sarcopenia may be reflected in poor liver function and higher mortality.

Three studies reported that patients with HCC had poor overall survival in a sarcopenia group than those without.^[7,11,12]^ The patients in these studies were treated by hepatectomy or RFA with potentially curable HCC. All of these studies measured all muscle volume by the cross-sectional area of skeletal muscle mass at the level of the middle of the third lumbar vertebrae. Yuri et al reported the association of low PMI and poor overall survival for HCC patients who had undergone RFA.^[8]^ Even though there were different CT methods used to measure muscle mass, previous studies revealed good correlation between total muscle area and psoas muscle area at the level of the middle of the third lumbar vertebra level.^[15]^ We also used the cut-off level of PMI from Japanese data, which is more suitable for our study than Western data.

A total of 47 patients expired during follow-up in this study. Up to 56% of our patients died due to liver disease or HCC (15 patients died due to liver failure, 4 patients due to HCC progression, 2 patients due to HCC rupture, 3 patients due to spontaneous bacterial peritonitis, and one patient due to gastric ulcer bleeding). Pre-sarcopenia might imply more severe liver disease and higher mortality even in patients with very early or early HCC. Adequate nutrition supplementation (especially branched-chain amino acids) and physical exercise might be effective treatment options for sarcopenia.

Previous studies revealed some prognostic factors such as gender, HBV, or HCV do not affect post-RFA recurrence. Tumor sizes and tumor number are the major parameters for predicting recurrence after RFA.^[10]^ However, few studies revealed these 2 parameters could also predict recurrence after RFA in very early- or early-stage HCC. Our studies revealed there was no prognostic factor for HCC recurrence after RFA treatment. Most studies revealed that sarcopenia is not associated with HCC recurrence, although 2 studies showed that patients with sarcopenia had higher recurrence rates than patients without sarcopenia and obesity (the cutoff levels of body mass index for obesity in these 2 studies were 22  and 25 kg/m^2^ respectively).^[7,13]^ These 2 studies enrolled some patients with BCLC stage B or C or surgical resection. One studies revealed there were no differences in HCC recurrence between sarcopenic and non-sarcopenic patients after stratification for body mass index.^[12]^ Intermediate or advanced stage and big tumor sizes are the only prognostic factors associated with disease-free survival. When comparing our study, we focused on the patients with very early- or early-stage HCC, *which means that there are few cases of intrahepatic metastasis or multicentre HCC before treatment.*

There are some limitations to our study. Firstly, we could only detect muscle mass in our patients because this was a retrospective study. We did not measure muscle strength modalities such as gait speed or handgrip strength to make a diagnosis of sarcopenia; however, patients with muscle mass loss might potentially have muscle strength decline. Further prospective studies might measure both muscle strength and muscle mass to identify patients with sarcopenia. Secondly, we did not have the cut-off value of PMI for sarcopenia in our domestic data. We used the data from a previous study in Japan as Asian data. However, there might be some small difference in cutoff level in different areas.

In summary, our study showed that patients with pre-sarcopenia had poor overall survival. Pre-sarcopenia was the independent prognostic factor for OS in patients with naïve HCC who underwent RFA. No prognostic factor for HCC recurrence of early HCC was found in this study. It might be important to survey the muscle depletion in patients with HCC for whom overall survival might be improved with intervention. Whether therapy of pre-sarcopenia could improve the outcomes in patients with early stage HCC or otherwise, should be evaluated in further prospective studies.

## Acknowledgments

We thank the staff of the Biostatistics Center, Kaohsiung Chang Gung Memorial Hospital, for statistics work.

## Author contributions

**Data curation and flow chart compilation:** Yeh WS, Chiang PL.

**Formal analysis:** Kee KM.

**Methodology:** Chang CD.

**Supervision:** Chen CH, Lu SN.

**Writing – original draft:** Yeh WS.

**Writing – review & editing:** Wang JH.
